# Phenotypes of *GNAO1* Variants in a Chinese Cohort

**DOI:** 10.3389/fneur.2021.662162

**Published:** 2021-05-28

**Authors:** Xiaoling Yang, Xueyang Niu, Ying Yang, Miaomiao Cheng, Jing Zhang, Jiaoyang Chen, Zhixian Yang, Yuehua Zhang

**Affiliations:** Department of Pediatrics, Peking University First Hospital, Beijing, China

**Keywords:** *GNAO1*, variant, epilepsy, movement disorder, developmental delay

## Abstract

This study aimed to analyze the genotypes and phenotypes of *GNAO1* variants in a Chinese cohort. Seven male and four female patients with *GNAO1* variants were enrolled, including siblings of brothers. Ten different *GNAO1* variants (nine missense and one splicing site) were identified, among which six were novel. All the variants were confirmed to be *de novo* in peripheral blood DNA. Eight (73%, 8/11) patients had epilepsy; the seizure onset age ranged from 6 h after birth to 4 months (median age, 2.5 months). Focal seizures were observed in all eight patients, epileptic spasms occurred in six (75%, 6/8), tonic spasm in four (50%, 4/8), tonic seizures in two, atypical absence in one, and generalized tonic–clonic seizures in one. Seven patients had multiple seizure types. Eight (73%, 8/11) patients had movement disorders, seven of them having only dystonia, and one having dystonia with choreoathetosis. Varying degrees of developmental delay (DD) were present in all 11 patients. The phenotypes were diagnosed as early infantile epileptic encephalopathy (EIEE) in two (18%) patients, which were further diagnosed as West syndrome. Movement disorders (MD) with developmental delay were diagnosed in two (18%) brothers. EIEE and MD were overlapped in six (55%) patients, among which two were diagnosed with West syndrome, one with Ohtahara syndrome, and the other three with non-specific EIEE. One (9%) patient was diagnosed as DD alone. The onset age of *GNAO1*-related disorders was early infancy. The phenotypic spectrum of *GNAO1* included EIEE, MD with DD, and DD alone.

## Introduction

In 2013, Nakamura et al. had reported, for the first time, the identification of *de novo* mutations in the *GNAO1* gene of four patients with early infantile epileptic encephalopathy-17 (EIEE17; 615473), and the mutations led to loss of function (1). In 2016, Ananth et al. reported *GNAO1* mutations in patients of neurodevelopmental disorder with involuntary movements (NEDIM; 617493) (2). In 2017, Feng et al. demonstrated that the epileptic encephalopathy phenotype is related more to *GNAO1* loss-of-function variants, while the movement disorders (MD), with or without epilepsy, were predominantly related to the gain-of-function variants (3).

The *GNAO1* gene encodes the α-subunit of the heterotrimeric guanine nucleotide-binding proteins (Gα_o_). G proteins are composed of α, β, and γ subunits. They are highly abundant in the mammalian central nervous system and are involved in signal transduction. They mediate signals from a variety of important G protein-coupled receptors (GPCRs) including GABAB, α2 adrenergic, adenosine A1 (A1R), and dopamine D2 (D2R) receptors (4). Mutations in *GNAO1* and other G-protein subunits (GNAL), adenylyl cyclase (ADCY5), and cyclic nucleotide phosphodiesterase (PDE10A) had been reported previously for early-onset movement disorders (5, 6). The G-protein–cAMP pathway axis is known to play a key role in the pathophysiology of movement disorders. At cellular level, Gα_o_ works as an inhibitor of voltage-gated Ca^2+^ channels and activator of inward rectifying potassium channels (7). Knockout of Gα_o_ in mice displayed complex variable neurological phenotypes including tremor, severe epilepsy, abnormal behavior, and severe motor control impairment (8).

In this study, we aimed to investigate the patients with *GNAO1* variants in a Chinese cohort, and explore the genotype–phenotype correlation for *GNAO1*.

## Materials and Methods

### Participants

Patients were enrolled from the Department of Pediatrics, Peking University First Hospital, between January 2016 and August 2020. Eleven patients with pathogenic *GNAO1* variants were collected and identified retrospectively. Written informed consent was obtained from the participants or their legal guardians for the publication of any potentially identifiable images or data included in this article. Information regarding seizure onset age, clinical manifestations, family history, genetic testing data, video electroencephalography (EEG), neuroimaging, and treatment were collected. Patients were followed-up at the pediatric neurology clinic or *via* telephone. The study was approved by the Ethics Committee of Peking University First Hospital [No: 2012 (453)]. Developmental delay of the patients was evaluated according to the developmental milestones at last follow-up.

The inclusion criteria were as follows: (a) genetic epilepsy with or without movement disorders, and (b) the proband carried pathogenic *GNAO1* variants in peripheral blood DNA as per Sanger sequencing. The exclusion criteria were as follows: (a) other causes of epilepsy, such as structural, metabolic, immune, and infectious, excluding genetic factor; and (b) absence of *GNAO1* variant in the peripheral blood DNA of probands.

### Genetic Analysis

Mutation screening of *GNAO1* (RefSeq NM_020988) was performed using epilepsy panel or whole-exome sequencing (WES). Synonymous changes and single-nucleotide polymorphisms (SNPs) with a minor allele frequency (MAF) higher than 5% were removed (http://www.ncbi.nlm.nih.gov/projects/SNP) from the sequencing results first. Pathogenicity of the identified variants was predicted by the MutationTaster server (http://www.mutationtaster.org/), Polymorphism Phenotyping v2 (PolyPhen-2) (http://genetics.bwh.harvard.edu/pph2/), and Sorting Intolerant from Tolerant (SIFT) (http://sift.jcvi.org/). *GNAO1* variants identified in the patients were evaluated against those in the literature, ClinVar, 1000 Genomes, ExAC (http://exac.broadinstitute.org/), and gnomAD (http://gnomad.broadinstitute.org/). Variant classifications were determined according to the guidelines set out by the American College of Medical Genetics and Genomics (ACMGG). The potential pathogenic variations were validated by Sanger sequencing.

## Results

### Genetic Variants

**Figure 1:**
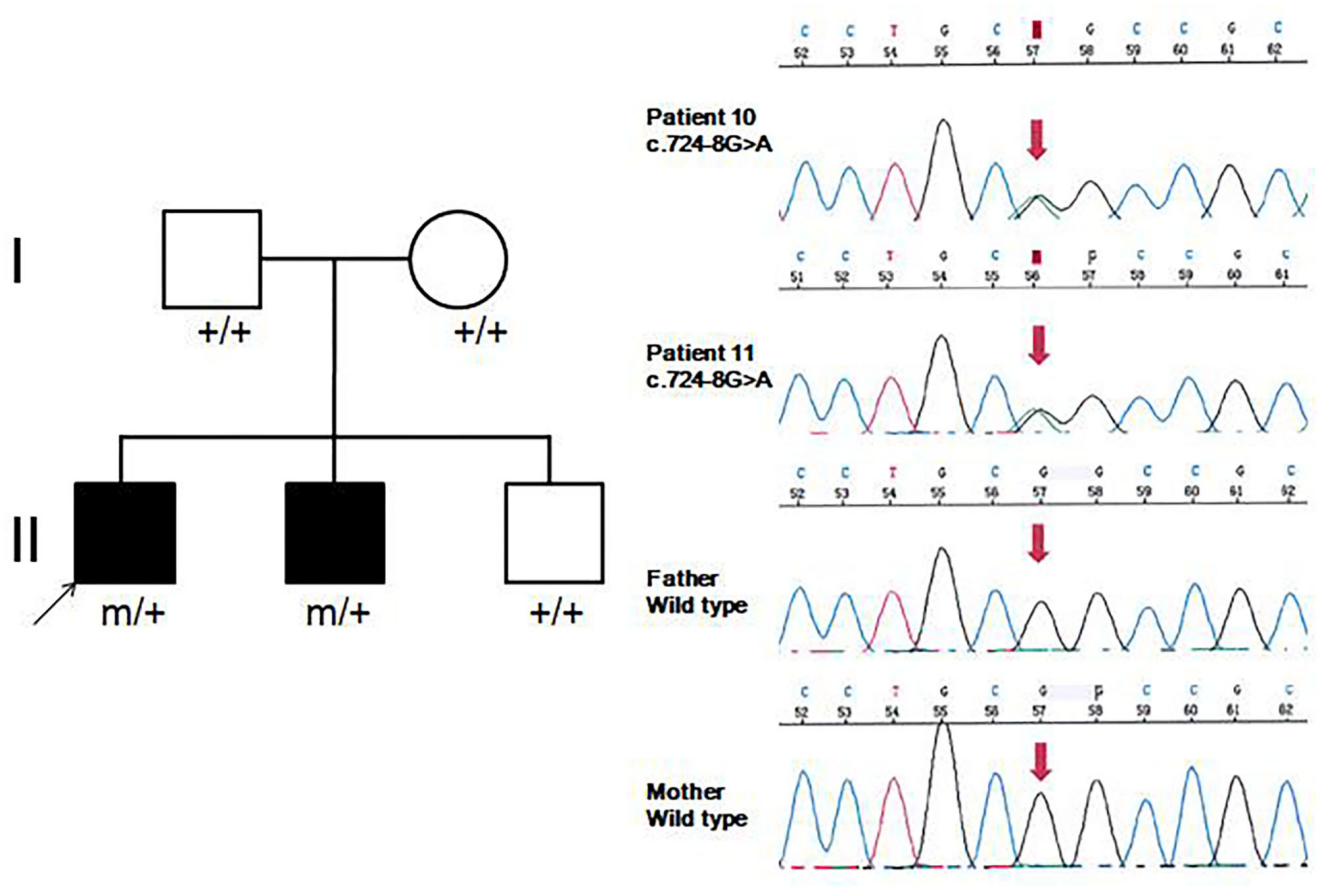
Family pedigrees and sequence chromatograms of the two brothers. The proband and his younger brother (Patients 10 and 11) were both identified with the c.724-8G>A variant, which was further verified as *de novo* in peripheral blood DNA.

**Figure 2:**
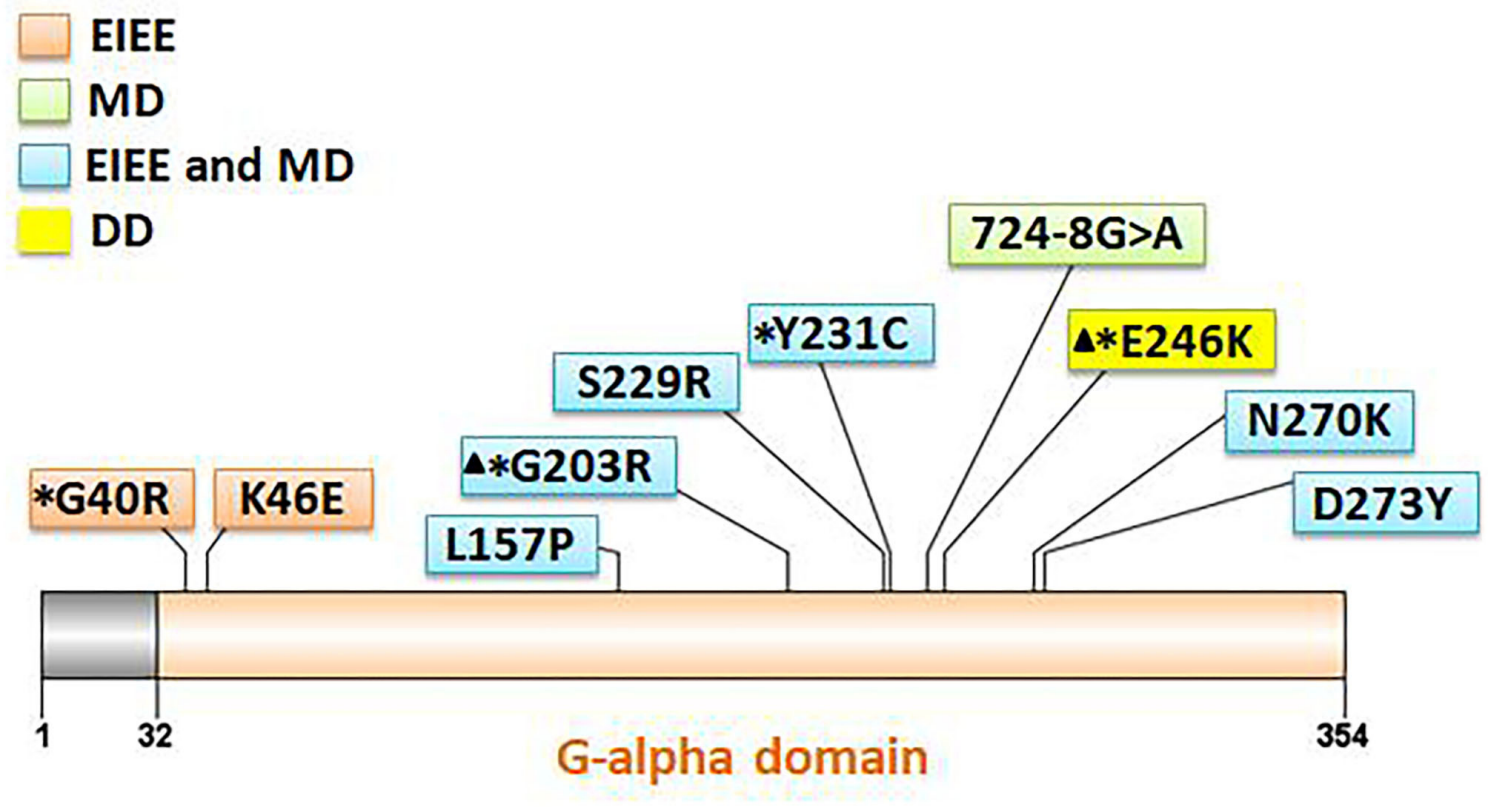
Location of 10 *GNAO1* variants in the Gαo amino acid sequence. All variants are indicated corresponding to their locations within the gene. Variants with asterisks had been reported previously, and those with triangles are hotspot variants. Different background color of the box represents different phenotypes (orange: EIEE, early infantile epileptic encephalopathy; green: MD, movement disorder; blue: EIEE and MD; yellow: DD, development delay).

Eleven patients with pathogenic *GNAO1* variants were collected in our cohort, of which seven were males and four females. Ten different pathogenic *GNAO1* variants were identified in the 11 patients; nine were missense and one was a splicing site variant. All the variants were verified as *de novo* in peripheral blood DNA after parental testing. Two brothers (Patients 10 and 11) shared the same splicing site variant c.724-8G>A (Figure 1). Six novel variants were detected, including c.136A>G (p.K46E), c.687C>G (p.S229R), c.470T>C (p.L157R), c.810C>A (p.N270K), c.817G>T (p.D273Y), and c.724-8G>A (Figure 2). The variants were predicted to be deleterious using the MutationTaster server, PolyPhen-2, and SIFT tools. The new variants had not been reported in 1000 Genomes, ExAC, or gnomAD previously. Details about the genotypes of all 11 patients are listed in Table 1.

**Table 1:** Genotypic and phenotypic features of 11 patients with *GNAO1* variants.

**Patient no**.	**Sex**	**Variant**	**Age at last follow-up**	**Onset age (symptom)**	**Seizure types**	**Movement disorders (symptom)**	**Developmental delay**	**EEG**	**MRI**	**Phenotypic diagnosis**	**Drugs**
1	F	c.136A>G (p.K46E)	1 y 11 m (death)	6 h (seizure)	FS, SS, TS, TSS	–	++	26 d: normal	Normal	West	VPA, TPM, PB, LEV, ACTH
								2 m 13 d: DSW, MFD			
								2 m 24 d: Hypsarrhythmia			
								11 m: SB, DSW, MFD			
2	M	c.687C>G (p.S229R)	1 y 9 m	1.5 m (seizure)	FS, SS, AAS	+(dystonia)	++	3 m, 5 m, and 1 y 1 m: Hypsarrhythmia	5 m: normal	West and MD	VPA, TPM, ACTH, VGB
								1 y 2 m: DSW of δ, MFD, temporal sharp wave	1 y 1 m: white matter delayed myelination		
3	F	c.470T>C (p.L157R)	1 y 5 m	1 d (seizure)	FS, SS, TSS	+(dystonia)	++	3 m: Hypsarrhythmia; few BS	Normal	West and MD	VGB, TPM, ACTH
4	M	c.118G>C (p.G40R)	1 y 1 m	4 m (seizure)	FS, SS	–	++	6 m: Hypsarrhythmia	4 m: wider extracerebral space	West	TPM, VPA, LEV
								1 y: SB, SSW of occiput			
5	M	c.810C>A (p.N270K)	1 y 7 m	9 d (seizure)	FS, SS, TS, TSS	+(dystonia)	++	1 m 19 d: BS mixed with few hypsarrhythmia	Normal	Ohtahara and MD	TPM, VPA, VGB, PER, LEV, LCM, PB, ACTH
								3 m 16 d and 4 m 13 d: BS			
6	F	c.817G>T (p.D273Y)	3 y 5 m	2 d (seizure)	FS, GTCS	+(dystonia)	++	1 y 1 m: DSW, occipital discharge	Normal	EIEE and MD	VPA, LEV, CZP, KD
								1 y 4 m: SB, right temporal discharge			
								1 y 6 m: Occipital discharge			
7	F	c.692A>G (p.Y231C)	8 m	3 d (seizure)	FS, SS, TSS	+(dystonia)	++	1 m: DSW	2 m: wider temporal extracerebral space	EIEE and MD	VPA, TPM, VGB
								2 m: parietal and occipital FW			
								3 m: occiput FWR, MFD			
8	M	c.607G>A (p.G203R)	10 m (death)	12 d (seizure)	FS	+(dystonia)	++	5 m: MFD	Normal	EIEE and MD	TPM, Levodopa
9	M	c.736G>A (p.E246R)	2 y 3 m (death)	4 m (DD)	–	–	++	4 m and 1 y 5 m: Normal	4 m: slightly wider frontotemporal extracerebral space	DD	–
									1 y 5 m: white matter developmental delay		
10	M	c.724-8G>A	16 y	5 m (DD)	–	+(dystonia, choreoathetosis)	+	7 y: SB	7 y: normal	MD and DD	Levodopa, CZP, Trihexyphenidyl
11	M	c.724-8G>A	7 y 3 m	4 m (DD)	–	+(dystonia)	+	3 y: SB	1 y 4 m: normal	MD and DD	Levodopa

*F, female; M, male; y, year; m, month; d, day; h, hour; DD, developmental delay; FS, focal seizures; TS, tonic seizures; SS, spasm seizures; TSS, tonic spasm seizures; AAS, atypical absence seizures; GTCS, generalized tonic-clonic seizures; DSW, diffuse slow wave; SSW, sharp slow wave; FW, fast wave; FWR, fast wave rhythm; MFD, multifocal discharge; SB, slow background; BS, burst-suppression pattern; EIEE, early infantile epileptic encephalopathy; MD, movement disorders; West, West syndrome; Ohtahara, Ohtahara syndrome; VPA, valproic acid; TPM, topiramate; PB, phenobarbital; PER, perampanel; LEV, levetiracetam; LCM, lacosamide; VGB, vigabatrin; CZP, clonazepam; KD, ketogenic diet; ACTH, adrenocorticotropic hormone*.

### Clinical Features

In 11 patients with *GNAO1* variants, eight (73%, 8/11) had epilepsy, eight (73%, 8/11) had movement disorders, and six (55%, 6/11) had both; all patients had developmental delay. Detailed information of clinical data is listed in Table 1.

In the eight patients with epilepsy, seizure onset age ranged from 6 h after birth to 4 months (median age, 2.5 months). While focal seizures were observed in all eight patients, epileptic spasms were seen in six (75%, 6/8), tonic spasm in four (50%, 4/8), and tonic seizures in two. Atypical absence seizures were observed in Patient 2. Generalized tonic–clonic seizures were observed in Patient 6. Seven patients had multiple seizure types. Status epilepticus was not observed in the patients.

Eight patients had clinical manifestation of movement disorders, including dystonia in all (Patients 2, 3, 5–8, 10, and 11), and choreoathetosis in one (Patient 10). Five patients (Patients 2, 3, and 5–7) had brief focal or truncal dystonia triggered by sound stimulus or emotional disturbance. Three patients (Patients 8, 10, and 11) had severe and persistent involuntary movements. Patients 10 and 11 were brothers, harboring the same *de novo GNAO1* variant c.724-8G>A (Figure 1). Patient 10 was born as the first child of healthy, non-consanguineous parents; at 5 months of age, motor developmental delay was observed by the parents. He had difficulties in head control and turning over. Independent walking was achieved at the age of 2 years, and he would walk sometimes unstably with tumble. He subsequently developed involuntary dystonia and choreoathetosis of the limbs. He had abnormal posture while walking. Symptoms disappeared when he was asleep. He had dysarthrosis and could not speak even at 16 years of age, as per last follow-up. His intellectual delay was mild. His pediatric neurologist had diagnosed him with movement disorders combined with developmental delay. His younger brother (Patient 11) also exhibited motor developmental delay at 4 months of age. He had dystonia at 3 years of age, although no choreoathetosis was seen at last follow-up at the age of 7 years.

All patients had developmental delay and hypotonia. Among the eight patients with epilepsy, the degree of DD was significantly severe in both motor and intellectual disabilities. They could not raise their head stably, or roll over, rarely grasped objects, and had little or no response to sound or light stimuli. In the two brothers (Patients 10 and 11) with MD, motor and intellectual delay was mild. Patient 9 had no epilepsy and movement disorder. He only manifested as DD, and had severe delayed milestones and intellectual disabilities.

**Figure 3:**
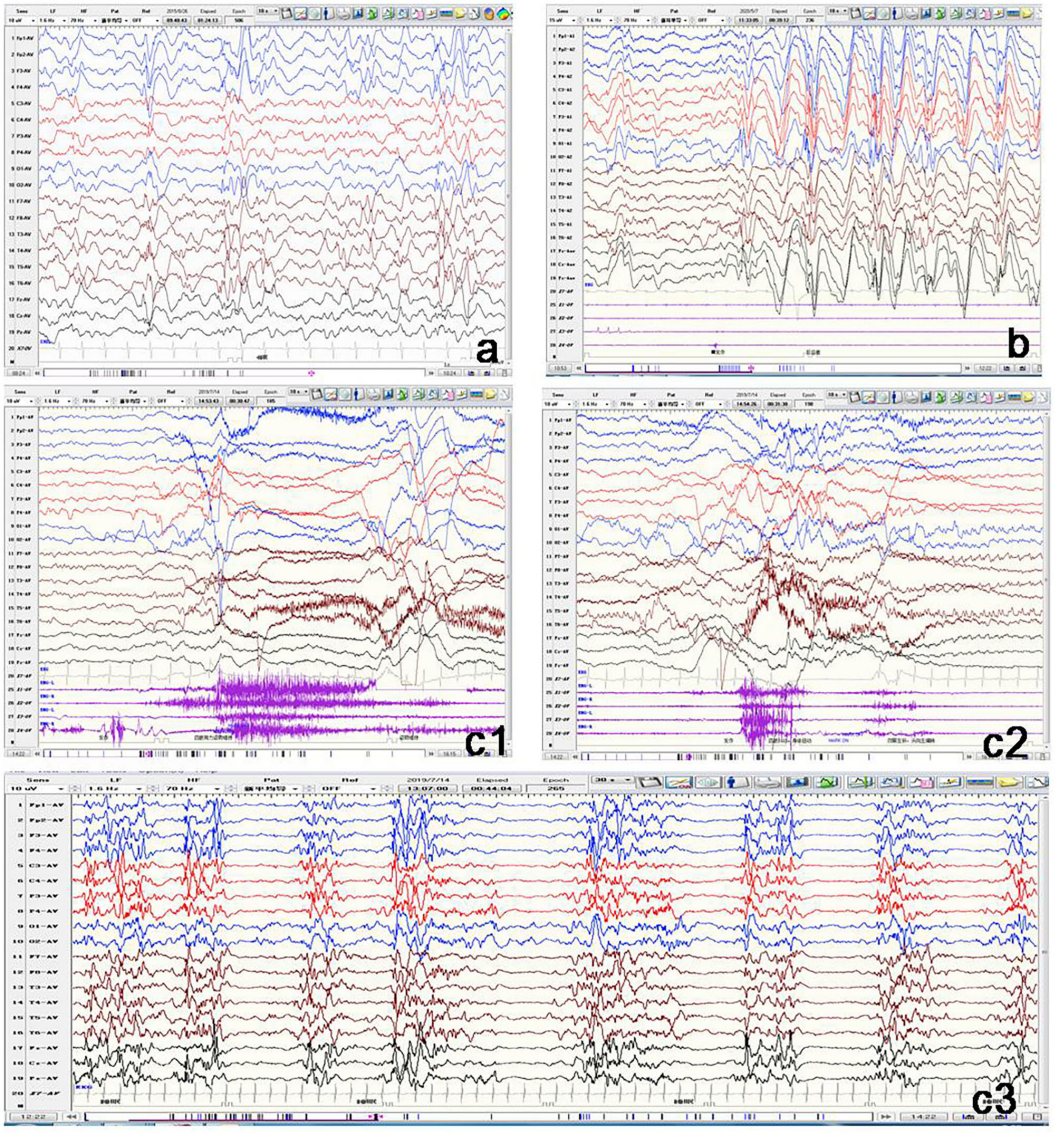
Representative electroencephalogram (EEG) of three patients with *GNAO1* variants. **(a)** Patient 1: Interictal EEG showing hypsarrhythmia. **(b)** Patient 2: Ictal EEG of atypical absence seizures monitored by generalized high-amplitude 1.5 Hz spike slow wave for several seconds. **(c)** Patient 5: c1: Ictal EEG of tonic spasm seizures showing widespread high-amplitude sharp wave, slow wave, and fast wave with a broad voltage reduction lasting several seconds. c2: Ictal EEG of focal seizures initiated from left occipital and posterior temporal regions. c3: Interictal EEG demonstrating suppression-burst pattern.

All the 11 patients underwent video EEG monitoring at least once. Out of the eight patients with epilepsy, EEG results showed three (Patients 1, 4, and 6) to have slow rhythm of background activity. All the eight patients with epilepsy had epileptiform discharges during the interictal phase (Figure 3). Hypsarrhythmia was observed in five patients (Patients 1–5). Two (Patients 3 and 5) revealed a suppression-burst pattern. Focal or multifocal epileptiform discharges were detected in all eight patients with epilepsy. Clinical seizures were recorded in six out of eight patients. Epileptic spasms and tonic spasms were recorded in four patients. Focal seizures were monitored in two patients. Tonic, generalized tonic–clonic, and atypical absence seizures were monitored in each patient. In Patient 9 with developmental delay only, EEG was normal. In the two brothers (Patients 10 and 11), EEG showed slow background activity, and no epileptic discharge.

All patients had undergone brain MRI. Their age at the time of MRI ranged from 1 month to 7 years. In Patient 3, brain MRI displayed white matter myelination delay at 1 year and 5 months of age. Slightly wider bilateral frontotemporal extracerebral space occurred in three patients (Patients 4, 7, and 9). White matter developmental delay was also observed in Patient 9. Brain MRI results were normal in a total of seven patients (64%, 7/11).

The phenotypes were diagnosed as EIEE in two (18%, 2/11) patients (Patients 1 and 4) and were further classified as West syndrome. The two brothers (18%, 2/11) were diagnosed with MD with DD. EIEE and MD were overlapped in six (55%, 6/11) patients, among which two were diagnosed with West syndrome (Patients 2 and 3), one with Ohtahara syndrome (Patient 5), and another three with non-specific EIEE (Patients 6, 7, and 8). One (9%, 1/11) patient was diagnosed with developmental delay only (Patient 9).

In the last follow-up of, eight patients had epilepsy, seven had tried two or more antiepileptic medications, and one (Patient 8) used only monotherapy. Seizures were well-controlled for 3–7 months in five patients. Five patients (Patients 3, 4, and 6–8) out of eight patients were seizure-free. Seizures were well-controlled with single topiramate in Patients 4 and 8, single vigabatrin in Patient 3, add-on therapy of levetiracetam in Patient 6, and add-on therapy of vigabatrin in Patient 7. Of the eight patients with MD symptoms, three (Patients 8, 10, and 11) were treated with levodopa, trihexyphenidyl, or clonazepam, but did not respond very well, and involuntary movements persisted. However, the hypertonia of limbs was mitigated to some extent. The last follow-up age of our cohort ranged from 8 months to 16 years. Three patients (27%) died before the last follow-up. Patient 1 died at 1 year and 11 months during sleep, presumably due to sudden unexpected death in epilepsy. Patient 8 died of milk choking and asphyxia at 10 months. Patient 9 died due to severe pneumonia and respiratory failure at the age of 2 years and 3 months.

## Discussion

Since 2013, *GNAO1*-associated neurological disorders have been consistently reported. *GNAO1* variants could cause multiple neurodevelopmental phenotypes, including epileptic encephalopathy, involuntary movements, and developmental delay. Kelly et al. had reported 14 patients with *GNAO1* variants, among which nine (64%) had epilepsy, nine (64%) had movement disorders, and five (36%) had both symptoms (9). Movement disorders included chorea, dystonia, dyskinesia, stereotypes, and ataxia. One patient with p.R209C only manifested developmental delay and hypotonia, but no seizure and movement disability. In our cohort of 11 patients, eight (73%) had epilepsy, eight (73%) had movement abnormalities, and six (55%) had both. One patient displayed developmental delay alone. All our patients had varying degrees of DD manifestations. *GNAO1*-related neurological phenotypes were of broad spectrum, including epilepsy, movement disorders, developmental delay, and combinations of all phenotypes.

Approximately 25 different *GNAO1* variants had been reported previously from more than 50 patients, including 23 (92%) missense, one frameshift deletion, and one splicing site variant (10). There are three mutation hotspots (G203, R209, and E246) in the *GNAO1* gene. A genotype–phenotype correlation was also noted recently (8). Many functional studies had been performed, especially for recurrent variants. Gain-of-function variants turned out to be associated with movement disorder, while loss-of-function variants were more related to epilepsy phenotype (3). However, the relationship of variant functionality with phenotype could not explain all the cases. The explicit genotype–phenotype correlations and mechanistic basis for heterogeneity still remain to be clarified. Further studies about phenotypes, genotypes, and molecular pathways would be required for evaluating the possible genotype–phenotype correlations better. In our current study, 10 different pathogenic *GNAO1* variants were identified, of which nine (90%) were missense, and one was a splicing site variant. All were verified as *de novo* in peripheral blood DNA. In previous reports, siblings sharing the same *GNAO1* variant and parental mosaicism of this gene had been confirmed in a few families (9, 11, 12). WES sequencing, performed for both siblings and their parents revealed a pathogenic variant in *GNAO1* at c.119G>A (p.G40E) that appeared to be *de novo* in each sibling. However, re-evaluation of parental WES data revealed three variant alleles out of 150 reads (2%) in one parent, consistent with germline mosaicism (9). Amplicon sequencing verified maternal mosaicism in one family. In this family, the patient carried the variant p.R209H, and Sanger sequencing analysis revealed a low heterozygous peak of the mother. This verification was crucial for genetic counseling in this family (12). In our study, the same variant (c.724-8G>A) was identified in siblings (Patients 10 and 11). We highly suspected parental germline mosaicism with low-level frequency. Further amplicon-deep sequencing detection for the mosaic variant frequency in multiple tissue samples needs to be performed in the future. This would be very important for genetic counseling.

Epilepsy is the major phenotype of the *GNAO1* gene variant. Previous studies had reported the onset of seizures to either precede or follow the MD manifestation (13). In the 22 patients reported with epilepsy (9), median age of seizure onset was 5 months (range, 1 month to 10 years); nine patients presented with EIEE, including two cases of Ohtahara syndrome and two of West syndrome. Seven patients exhibited focal seizures, while three had generalized tonic–clonic seizures. In the remaining three patients, the epileptic syndrome was not specified. In our eight patients with epilepsy, the age of seizure onset ranged from 6 h after birth to 4 months. Multiple seizure types were observed in most patients. The common seizure types were focal and epileptic spasms in our patients. The epileptic syndromes included West syndrome, Ohtahara syndrome, and non-specific EIEE. Feng et al. had reported that seizures were well-controlled by medication in half of the patients with epilepsy and *GNAO1* variants (10). Responses to topiramate and levetiracetam had been reported earlier (10). Among our eight patients with epilepsy, seizures were completely controlled in five by topiramate, vigabatrin, and levetiracetam treatment. Results from published literature and our current study suggested that treatment with topiramate, levetiracetam, or vigabatrin should be recommended for patients with epilepsy and *GNAO1* variants.

Movement abnormalities were also the major presenting symptoms of patients with *GNAO1* variants. The median age at MD onset was 4 years, ranging from 3 months to 8 years, as per published reports (13). Most patients had a mixed hyperkinetic syndrome, encompassing different combinations of dystonia, choreoathetosis, and dyskinetic movements (13). In the overall cohort, dystonic features were most frequently reported to be a component of the MD (65%, 30/46), followed by dyskinesias (63%, 29/46) and choreoathetosis (58%, 27/46). Eight patients in our cohort had MD; its range included dystonia and choreoathetosis. The most common feature of MD in our patients was dystonia. The last follow-up age of our patients was <4 years, and we believe that they should be followed-up further for their clinical manifestations, including movement disorders. EEG showed low background activity but no abnormal discharge in patients with only MD (Patients 10 and 11). Partial patients obtained satisfactory management of the MD with oral drugs, the effects being variable across the different cases. They were treated with phenobarbital, benzodiazepines (clonazepam and diazepam), levodopa, neuroleptics (haloperidol and risperidone), and antiepileptics (topiramate, carbamazepine, valproate) (2, 11, 14–16). In one case of severe MD with the *GNAO1* variant, the episodes were treated successfully with gabapentin (17). However, few patients presenting with severely medical-refractory MD required surgical treatment. Deep brain stimulation (DBS) was performed to reduce the frequency and severity of MD exacerbations (13). Three of our patients were administered oral drugs of levodopa, trihexyphenidyl, or clonazepam for controlling the MD symptoms; however, the effects were not satisfactory. DBS may be suggested and tried in such patients with refractory persistent dystonia.

Together with epilepsy or involuntary movements, the phenotypes involved global retardation of motor and intellectual development in patients with *GNAO1* variants. Kelly et al. reported all 14 patients to have developmental delay and show a broad spectrum of severity (9). Five (36%) patients showed profound impairment and were non-verbal and non-ambulatory. Few patients had the mildest developmental abnormalities, including delayed speech and motor development and mild intellectual disability. All our 11 patients had developmental delay ranging from mild to severe. In patients with epilepsy, the developmental disability was global retardation involving motor and intellectual delay. The progress was still slow in patients with controlled seizure. In two patients with MD and seizure unaffected, their motor developmental difficulties were significantly severe, and intellectual delay was milder. In our cohort, Patient 9 with *GNAO1* variant, p.E246K had the phenotype of DD alone. Age at the last follow-up was 2 years and 3 months. No symptom of seizure and movement disorder was observed in this patient, although the global developmental delay was significantly severe. Regrettably, he died due to respiratory failure and sepsis after pulmonary infection. Twelve patients with the variant p.E246K had been reported previously (2, 11, 18), and the variant was located in a mutational hot spot. For available detailed information in eight patients, seven patients had MD, and only one patient had focal epilepsy. The data led to the speculation that the p.E246K variant may be a specific site for MD phenotypes, usually without epilepsy.

The phenotypic spectrum of *GNAO1*-related neurodevelopmental disorders ranges broadly from epileptic encephalopathy, movement disorders, and developmental delay, to the combination of various phenotypes. *De novo* missense variants were frequent in the *GNAO1* gene. The age of seizure onset, in the presence of the *GNAO1* variant was early infancy. Dystonia may be the most common symptom related to movement disorders. When patients with EIEE had combined dystonia, *GNAO1* may be suspected as the causative gene. All patients had developmental delay with a wide range of severity; however, the genotype–phenotype correlations are still unclear. Further studies on genotype, phenotype, and molecular pathways would be required for better understanding of the process.

## Data Availability Statement

The datasets presented in this study can be found in online repositories. The names of the repository and accession numbers can be found below: ClinVar database and SCV001486211—SCV001486219, SCV001499870.

## Ethics Statement

The studies involving human participants were reviewed and approved by the Ethics Committee of Peking University First Hospital [No: 2012 (453)]. Written informed consent to participate in this study was provided by the participants' legal guardian/next of kin. Written informed consent was obtained from the individual(s), and minor(s)' legal guardian/next of kin, for the publication of any potentially identifiable images or data included in this article.

## Author Contributions

XY and YZ jointly conceived the study and contributed to the study design, initial writing, and revision of the manuscript. XN, YY, MC, JZ, JC, ZY, and YZ collected the patient data. All authors contributed to the article and approved the submitted version.

## Conflict of Interest

The authors declare that the research was conducted in the absence of any commercial or financial relationships that could be construed as a potential conflict of interest.
